# Danshen extract circumvents drug resistance and represses cell growth in human oral cancer cells

**DOI:** 10.1186/s12906-017-2063-y

**Published:** 2017-12-29

**Authors:** Cheng-Yu Yang, Cheng-Chih Hsieh, Chih-Kung Lin, Chun-Shu Lin, Bo Peng, Gu-Jiun Lin, Huey-Kang Sytwu, Wen-Liang Chang, Yuan-Wu Chen

**Affiliations:** 1 School of Dentistry, National Defense Medical Center, Taipei, Taiwan, Republic of China; 2 Department of Pharmacy Practice, Tri-Service General Hospital, Taipei, Taiwan, Republic of China; 3 Division of Anatomic Pathology, Taipei Tzu Chi Hospital, Taipei, Taiwan, Republic of China; 4 Department of Radiation Oncology, Tri-Service General Hospital, National Defense Medical Centre, Taipei, Taiwan, Republic of China; 5 Graduate Institute of Clinical Medicine, College of Medicine, Taipei Medical University, Taipei, Taiwan, Republic of China; 6 Department of Biology and Anatomy, National Defense Medical Center, Taipei, Taiwan, Republic of China; 7 Graduate Institute of Microbiology and Immunology, National Defense Medical Center, Taipei, Taiwan, Republic of China; 8 School of Pharmacy, National Defense Medical Center, Taipei, Taiwan, Republic of China; 9 Department of Oral and Maxillofacial Surgery, Tri-Service General Hospital, No. 161, Section 6, Min-Chuan East Road, Neihu 114, Taipei, 114 Taiwan, Republic of China

**Keywords:** Danshen, Oral cancer, Drug resistance, Apoptosis

## Abstract

**Background:**

Danshen is a common traditional Chinese medicine used to treat neoplastic and chronic inflammatory diseases in China. However, the effects of Danshen on human oral cancer cells remain relatively unknown. This study investigated the antiproliferative effects of a Danshen extract on human oral cancer SAS, SCC25, OEC-M1, and KB drug-resistant cell lines and elucidated the possible underlying mechanism.

**Methods:**

We investigated the anticancer potential of the Danshen extract in human oral cancer cell lines and an in vivo oral cancer xenograft mouse model. The expression of apoptosis-related molecules was evaluated through Western blotting, and the concentration of in vivo apoptotic markers was measured using immunohistochemical staining. The antitumor effects of 5-fluorouracil and the Danshen extract were compared.

**Results:**

Cell proliferation assays revealed that the Danshen extract strongly inhibited oral cancer cell proliferation. Cell morphology studies revealed that the Danshen extract inhibited the growth of SAS, SCC25, and OEC-M1 cells by inducing apoptosis. The Flow cytometric analysis indicated that the Danshen extract induced cell cycle G0/G1 arrest. Immunoblotting analysis for the expression of active caspase-3 and X-linked inhibitor of apoptosis protein indicated that Danshen extract-induced apoptosis in human oral cancer SAS cells was mediated through the caspase pathway. Moreover, the Danshen extract significantly inhibited growth in the SAS xenograft mouse model. Furthermore, the Danshen extract circumvented drug resistance in KB drug-resistant oral cancer cells.

**Conclusion:**

The study results suggest that the Danshen extract could be a potential anticancer agent in oral cancer treatment.

## Background

Oral cancer is one of the most common cancers and the leading cause of cancer deaths worldwide [1]. Recent understanding of oral cancer has led to the development of biological therapies using drugs such as 5-fluorouracil (5-FU) and cisplatin [2]. These drugs have shown remarkable activity against difficult-to-treat oral cancer in early clinical trials; however, prolonged drug exposure may result in the development of de novo drug resistance and unexpected side effects, such as allergic reactions, breathing difficulties, swelling, nausea, fever or chills, and dizziness or weakness [3–5]. Therefore, the identification and validation of novel targeted therapies is urgently required to overcome drug resistance and improve patient outcomes.

Danshen (*Salvia miltiorrhiza*) is a widely used traditional Chinese medicine, which was first described in the Chinese pharmacology book, *Shen Nong’s Canon on Materia Medica* [6]. Danshen attenuates inflammatory reactions in cardiovascular, hepatic, and tumoral diseases without appreciable adverse effects [6]. Various Danshen extracts contain diterpene quinone and phenolic acid derivatives including tanshinone, cryptotanshinone, isocryptotanshinone, miltirone, tanshinol, salviol, and salvianolic acid B [7–9]. Because of their growth-inhibiting effects on cancer cells [7], Danshen extracts may be suitable as major drug candidates or additional chemotherapeutic agents in oral cancer treatment. In this study, we observed that a Danshen extract (crude) can inhibit human oral cancer SAS, SCC25, OEC-M1, and KB drug-resistant cell lines. It possibly exerts anticancer effects by blocking cell cycle entry into the G1 phase in oral cancer cells.

## Methods

### Reagents

In this study, 5-FU was purchased from Sigma-Aldrich (F6627); its purity was ≥99%, as determined by high-performance liquid chromatography. The 5-FU was dissolved in saline as a 1.5 mg/mL stock and used as the positive control in an animal model.

### Preparation and treatment of the Danshen extract

Danshen (*S. miltiorrhiza*) was obtained from Dr. Wen-Liang Chang of the National Defense Medical Center in Taipei, Taiwan [10, 11]. Danshen roots were obtained from Chien Yuan Herbal Medicinal Co., Taipei, Taiwan, and identified to be Salviae miltiorrhizae Radix. The pulverized roots (4.5 kg) were extracted with 95% ethanol (15 L) exhaustively for five times. The extract was concentrated by evaporation under reduced pressure. The dried extracts were dissolved in dimethylsulfoxide to prepare a 20 mg/mL stock solution and stored at 4 °C. The Danshen extract was diluted with culture media to achieve the indicated final concentration in each experiment.

### Cell culture

The human oral squamous cell carcinoma (OSCC) cell line SAS (JCRB0260) was purchased from the Japanese Collection of Research Bioresources Cell Bank. SCC25 (CRL-1628) was obtained from the American Type Culture Collection (ATCC). OEC-M1 cell line was derived from oral cavity epidermal carcinoma [12], which is a generous gift from Prof. Jenn-Han Chen (National Defense Medical Center, Taiwan). KB drug-resistant cancer cells were purchased from the ATCC (CCL-17; Rockville, MA, USA). KB-7D cells were generated through etoposide (VP-16)-driven selection, which demonstrated topoisomerase II downregulation and multidrug resistance-associated protein overexpression. KB-tax cells were generated through taxol-driven selection. These drug-resistant cancer cells were kindly provided by Dr. Jang-Yang Chang (Cancer Research Division of National Health Research Institutes, Taiwan) [13]. Human oral cancer SAS, SCC25, and OEC-M1 cells were cultured in Roswell Park Memorial Institute 1640 medium. The culture medium was supplemented with 10% fetal bovine serum, 1% penicillin/streptomycin, and 2 mmol/L L-glutamine. The cells were grown at 37 °C in a humidified 5% CO_2_ incubator.

### Cytotoxicity assay

The cells (10,000 cells/well) were cultured in a 24-well plate and then exposed to various concentrations of the Danshen extract for 24 h. The methylene blue dye assay was performed to evaluate the effects of melatonin on cell growth, as described previously [10]. The half-maximal inhibitory concentration (IC50) value resulting from 50% inhibition of cell growth was calculated graphically for comparison with cell growth in controls.

### Cell cycle analysis

The cells were harvested with 0.25% trypsin and washed once with phosphate-buffered saline (PBS). After centrifugation, the cells were fixed in 100% ice-cold methanol overnight at −20 °C; next, they were incubated in propidium iodide (50 μg/mL) and RNase (1 mg/mL) for 30 min. Apoptotic cells were identified using a FACScan flow cytometer (Becton Dickinson, Mountain View, CA, USA), and the data were analyzed using CellQuest software. All experiments were performed in triplicate.

### Western blot analysis for caspase activity

The cells were lysed directly in an radioimmunoprecipitation assay buffer containing 50 mM Tris (pH 7.8), 0.15 M NaCl, 5 mM ethylenediaminetetraacetic acid, 0.5% Triton X-100, 0.5% NP-40, 0.1% sodium deoxycholate, a protease inhibitor mixture (Calbiochem, Billerica, MA, USA), and a phosphatase inhibitor mixture (Calbiochem, USA). The relative protein concentration in supernatants was determined using a bicinchoninic acid protein assay kit (Thermo Scientific, Rockford, IL, USA). For immunoblotting, in each lane of 10% sodium dodecyl sulfate–polyacrylamide electrophoresis gel, 30 μg protein from cell lysates was loaded, separated, and transferred onto polyvinyldifluoride membranes (GE Healthcare, UK). These membranes were subsequently probed using specific antibodies against caspase-3 (Cell Signaling, #9662), X-linked inhibitor of apoptosis protein (XIAP; Cell Signaling, #2045), and glyceraldehyde-3-phosphate dehydrogenase (Epitomics, #2251-1).

### OSCC animal models

To investigate the effects of the Danshen extract on OSCC in vivo, we used oral cancer SAS xenograft animal models. All experiments were approved by the Institutional Animal Use Committee (IACUC) of the National Defense Medical Center, Taiwan (IACUC 16-022). Eight-week-old nonobese diabetic/severe combined immunodeficiency (NOD/SCID) (NOD.CB17 Prkdc scid/J, National Laboratory Animal Center, Taiwan) mice were maintained in microisolators under specific pathogen-free conditions. These NOD/SCID mice were fed with sterile food and chlorinated sterile water. A total of 12 mice were divided into 3 groups: Danshen extract (10 mg/kg body weight [BW]/d/intraperitoneally [i.p.])-treated; positive control (5-FU, 10 mg/kg BW/d/i.p.); and vehicle control (PBS). Each group of mice was subcutaneously injected with 2 × 10^6^ human oral cancer SAS cells. Drugs were first administered to each group of mice on day 3 prior to tumor palpation, and the treatment was continued until day 32. The size of the transplanted tumors was measured every 3 days using gauged calipers, and the tumor volume was calculated using the following formula: V = 1/2 × (length × width^2^). At the end of the treatment, the mice were sacrificed, and the tumors were removed, weighed, and photographed.

### Haemotoxylin and eosin and immunohistochemical staining

Mice were sacrificed with CO_2_ inhalation and fixed by perfusion with 4% paraformaldehyde in 0.1 M phosphate buffer. Serial 5-μm histologic sections were deparaffinized in xylene and rehydrated. After blocking of endogenous peroxidase by incubation with 3% hydrogen peroxide, the slides were incubated with anticaspase-3 overnight at 4 °C. The expression levels of targeted proteins were examined by using the antimouse and rabbit peroxidase complex, and peroxidase activity was evaluated using 3-amino-9-ethyl-carbazole. The slides were counterstained with hematoxylin (Sigma) and mounted with mounting solution. Scores were obtained by multiplying the immunohistochemical (IHC) intensity with the percentage of cancer cells stained.

### Statistical analysis

The experiments were performed in triplicate. The data for cell proliferation and viability assays are expressed as the mean ± standard deviation. Standard deviations for all measured biological parameters are displayed in the appropriate figures. The Student t test was performed to determine the significance of the differences between the control and treated groups for all experimental test conditions. *P* < 0.05 was considered statistically significant. Statistical analysis was performed using GraphPad Prism (GraphPad Software, San Diego, CA, USA).

## Results

**Fig. 1:**
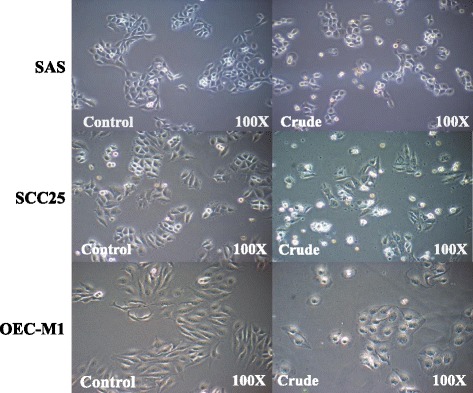
Danshen crude induced morphological change in oral cancer cells. Effect of the Danshen crude on morphological changes in SAS, SCC25, and OEC-M1 oral cancer cell lines. Original magnification, 100×

**Fig. 2:**
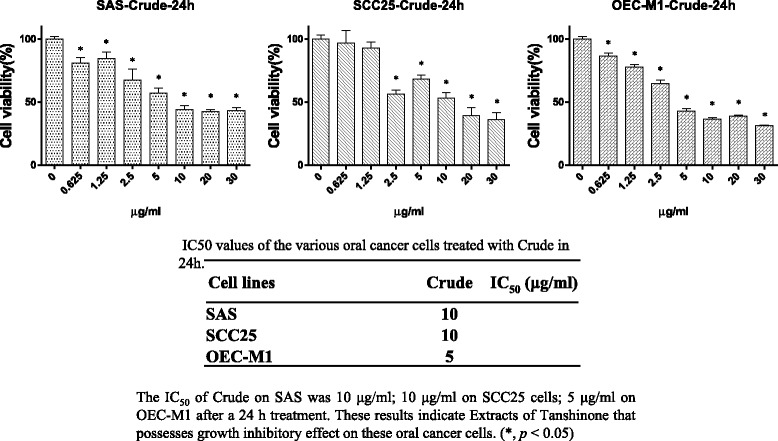
Inhibition of oral cancer cell (SAS, SCC25, and OEC-M1) growth in vitro via the Danshen crude. The antioral cancer efficacy of the Danshen crude on three oral cancer cell lines (SAS, SCC25, and OEC-M1) treated for 24 h. The extract significantly reduced the cell viability of all three OSCC lines in a dose-dependent manner

The growth inhibitory effects of a Danshen extract on human oral cancer SAS, SCC25, and OEC-M1 cells were first demonstrated by conducting microscopic studies. The results showed that the Danshen extract effectively inhibited the growth of both tongue cancer cells (SAS and SCC25) and gingival cancer cells (OEC-M1) (Fig. 1). The antiproliferative effects of the Danshen extract on human oral cancer cells were further confirmed by proliferation assays (Fig. 2). The methylene blue proliferation assay revealed that Danshen treatment (5–10 μg/mL) could result in an approximately 50% reduction in oral cancer cell proliferation (Fig. 2).

**Fig. 3:**
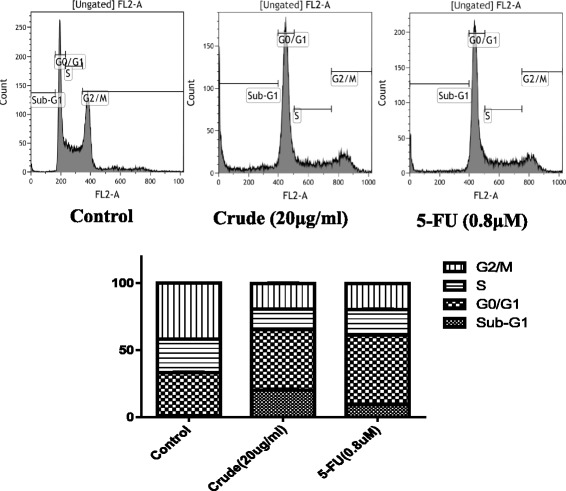
Oral cancer G0/G1 arrest induced by the Danshen crude. Flow cytometry analysis results of crude- and 5-FU-treated SAS cells. The cells were treated with extract (20 μg/mL) and 5-FU (0.8 μM) for 24 h. Harvested cells were stained with propidium iodide

To examine whether the Danshen extract inhibited cell proliferation by cell cycle arrest, cell cycle profiling of the SAS cells was performed. The cells were treated with the Danshen extract (20 μg/mL) for 24 h and their cell cycle profiles were analyzed using flow cytometry. The analysis results revealed that the Danshen extract engendered an increase in the G0/G1 phase, with a concomitant reduction in the S phase, in the SAS cells (Fig. 3). In addition, the sub-G1 apoptotic dead cell population increased after Danshen treatment (Fig. 3). These results suggest that the Danshen extract induced oral cancer cell apoptosis and cell cycle G0/G1 arrest.

**Fig. 4:**
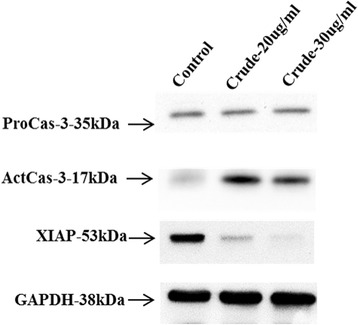
Apoptosis in oral cancer cells induced by the Danshen crude. Lysates of the SAS cells treated with extract (20 and 30 μg/mL) for 24 and 48 h were subjected to an immunoblot analysis with antibodies against caspase-3 and XIAP. GAPDH served as the loading control

To gain a clearer understanding of the antioral cancer mechanism in Danshen-extract-treated cancer cells, we investigated the effects of the Danshen extract on the expression levels and activities of intracellular signaling molecules (Fig. 4). Specifically, the expression levels of the apoptotic protein caspase-3 and antiapoptotic protein XIAP were upregulated and downregulated, respectively, in Danshen-extract-treated cancer cells (Fig. 4). To delineate the antiproliferative effects of the Danshen extract, the SAS cells were treated with various concentrations of the extract for different treatment periods. In addition, Western blotting was performed to examine the effects of the Danshen extract on intracellular signaling molecules. The results (Fig. 4) demonstrated that the Danshen extract effectively suppressed XIAP expression and upregulated caspase-3 expression.

**Fig. 5:**
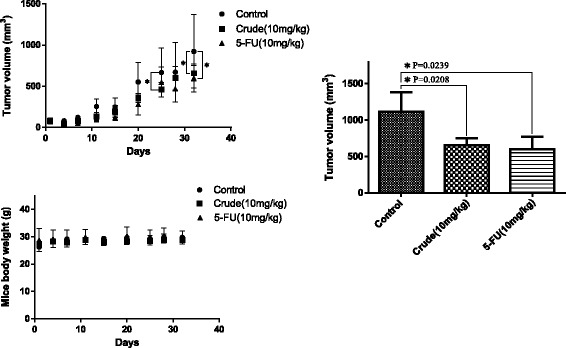
Repression of tumor cell growth in SAS xenograft mice via the Danshen crude. The Danshen extract inhibited oral cancer cell growth in vivo. The SAS xenograft was exposed to either saline (control) or the Danshen extract (crude, 10 mg/kg), and 5-FU (10 mg/kg) served as the positive control

The antioral cancer effects of the Danshen extract were further investigated in the SAS solid tumor xenografts of immunodeficient mice. The daily treatments were initiated a day after the SAS cells were transplanted into the NOD/SCID mice. The doses for individual treatments (for 32 d) are outlined as follows: Danshen extract, 10 mg/kg/d/i.p.; 5-FU, 10 mg/kg/d/i.p. During this period, each mouse was manually examined for tumor volume at least four times. The Danshen extract and 5-FU (i.e., the first-line drug for oral cancer treatment) significantly inhibited tumor growth in the NOD/SCID mice, and the bodies weight of the mice were not changed (Fig. 5).

**Fig. 6:**
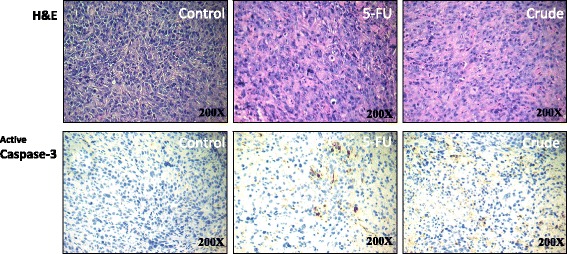
Active caspase-3 expression induced in oral cancer cells in the SAS xenografts model using the Danshen crude. H&E staining and IHC were performed after administration of the Danshen crude, 5-FU, or PBS (a vehicle control). The SAS xenografts model stained active caspase-3. Immunodetectable proteins are stained brown and the nuclei are counterstained blue. Original magnification, 200×

Histologic sections obtained from the SAS xenografts were used to evaluate the antiproliferative effects of the Danshen extract in SAS cells. The expression levels of caspase-3 (apoptotic biomarker) were then analyzed through IHC staining (Fig. 6), which our statistical analysis showed were significantly higher in the Danshen-extract-treated group than in the untreated control groups.

**Fig. 7:**
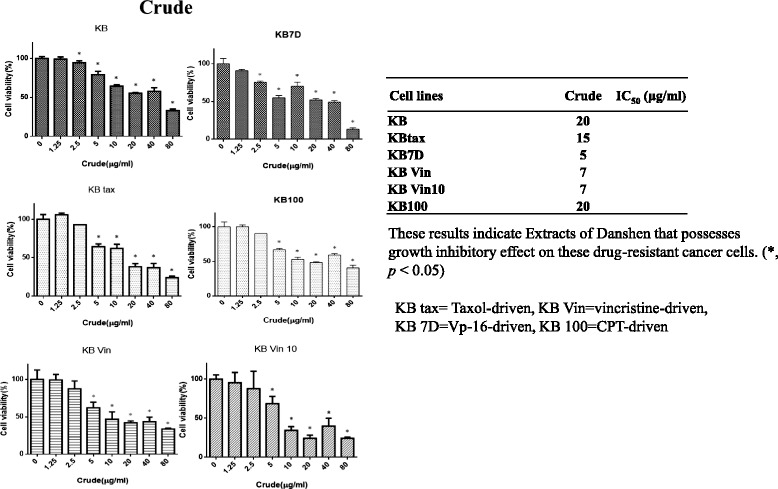
Inhibition of drug-resistant KB cell growth in vitro via the Danshen crude. An assessment of cell proliferation and viability by using the methylene blue assay in six drug-resistant oral cancer cells treated with varying concentrations of the Danshen crude (0–80 μg/mL) or dimethyl sulfoxide (1 μL/mL) for 24 h

The Danshen extract also significantly inhibited the proliferation of KB drug-resistant cells, indicating that the treatment had circumvented drug resistance in these cells (Fig. 7).

## Discussion

The prevalence of oral cancer has increased globally in recent years [14], and 5-FU-based chemotherapy has been widely used to reduce the risk of relapse after surgery. The 5-FU plus docetaxel treatment, with the addition of oxaliplatin chemotherapy (which improves survival significantly compared with 5-FU alone [15]), has been widely accepted as the standard adjuvant chemotherapy for OSCC. However, inflammation, neutropenia and lymphopenia, which are common chemotherapy-induced toxicities, may influence the prognosis of adjuvant chemotherapy in cancer treatment [16, 17]. By contrast, Danshen is a natural compound that has anti-inflammation and anticancer effects, which can be effective for the supportive care of cancer patients [18]. Some clinical studies have indicated that bioactive natural compounds play a key role in the treatment of many cancers [6, 19]. In the present study, a Danshen extract was observed to inhibit oral cancer cell proliferation (Figs. 1 and 2) through cell cycle G0/G1 arrest (Fig. 3).

Apoptosis is a well-defined self-suicidal process to inhibit tumor growth. Many studies have reported that chemotherapeutic drugs exert antitumor effects by triggering apoptosis through various molecular mechanisms [20]. One previous study revealed that tanshinone IIA induces apoptosis in human oral cancer KB cells through a mitochondria-dependent pathway [8]. Our results demonstrate that the Danshen extract upregulated caspase-3 expression and repressed XIAP expression in SAS cells. In addition, our results confirm the involvement of apoptosis in Danshen-induced in vitro and in vivo growth inhibition in human oral cancer cells (Figs. 4, 5 and 6).

Cell cycle dysregulation results in uncontrolled cell growth, which can lead to cancer development [21]. Therefore, the targeted detection of cell-cycle-related errors in cancer cells is considered a potential strategy for tumor growth control [22]. Research has revealed that the leading bioactive components (salvianolic acid B [23] and tanshinone IIA [8]) of Danshen result in increased G0/G1 and G2/M phases in oral cancer cells, and in head and neck cancer cells, respectively. The present study revealed cell cycle G0/G1 arrest in Danshen-extract-treated SAS cells and an increased sub-G1 apoptotic dead cell population (Fig. 3). These findings suggest that the Danshen extract induces cell cycle G0/G1 arrest and apoptosis.

Resistance to chemotherapy and molecular-targeted therapies is a major concern in current cancer research. Our results reveal that the Danshen extract suppressed growth in drug-resistant oral cancer cells (including taxol- [24], vincristine- [25], Vp-16- [26], and camptothecin- [27] resistant cells; Fig. 7). These results suggest that the Danshen extract may be a potential novel chemotherapeutic agent in oral cancer treatment.

## Conclusions

In conclusion, this in vitro and in vivo study revealed that a Danshen extract exerts antiproliferative effects in human oral cancer cells and KB drug-resistant cells through multiple pathways, such as the induction of apoptosis. Therefore, Danshen treatment should be considered a novel approach for drug-resistant oral cancer prevention and treatment.

## Abbreviations

NOD/SCID: Nonobese diabetic/severe combined immunodeficiency
OSCC: Oral squamous cell carcinoma
XIAP: X-linked inhibitor of apoptosis protein
